# Case Report: Discordant high-sensitivity cardiac troponin T elevation in polymyositis: a diagnostic pitfall

**DOI:** 10.3389/fcvm.2026.1854699

**Published:** 2026-07-23

**Authors:** Shuanglin Zheng, Huabin Xie

**Affiliations:** Department of Clinical Laboratory, Xiamen Cardiovascular Hospital, Xiamen University, Xiamen, China

**Keywords:** diagnostic pitfall, high-sensitivity cardiac troponin I, high-sensitivity cardiac troponin T, PEG precipitation, polymyositis, troponin discordance

## Abstract

**Background:**

High-sensitivity cardiac troponin T (hs-cTnT) is widely used as a gold-standard marker of myocardial injury, yet non-ischemic elevations can occur in idiopathic inflammatory myopathies (IIM), creating a significant diagnostic pitfall. In contrast, high-sensitivity cardiac troponin I (hs-cTnI) generally retains strict cardiac specificity.

**Case report:**

A 79-year-old female presented with progressive fatigue and elevated cardiac enzymes. Admission hs-cTnT was markedly elevated at 521 ng/L (reference 0–14 ng/L), and she was initially suspected of having a non-ST elevation myocardial infarction (NSTEMI) and underwent emergency coronary angiography, which revealed non-obstructive coronary artery disease. Polyethylene glycol (PEG) precipitation demonstrated a recovery rate exceeding 80%, excluding macromolecular complex interference, while follow-up hs-cTnT testing at an external hospital confirmed the elevation. However, hs-cTnI measured on both the Abbott and Yhlo platforms remained within normal limits. This hs-cTnT(+)/hs-cTnI(−) discrepancy, combined with electromyography (myogenic damage), muscle MRI, and a normal echocardiogram, established the final diagnosis of polymyositis (PM).

**Conclusion:**

Regenerating skeletal muscle fibers can re-express embryonic cTnT isoforms, producing a true circulating cTnT elevation of skeletal muscle origin that mimics myocardial injury. PEG precipitation confirmed authentic monomeric cTnT antigen rather than an analytical artifact, redirecting the diagnostic focus. Concurrent hs-cTnI assessment is a critical strategy for distinguishing skeletal muscle-derived cTnT from true myocardial injury, thereby preventing misdiagnosis and unnecessary interventions in patients with inflammatory myopathies.

## 1 Introduction

Cardiac troponin (cTn) is the cornerstone biomarker for the diagnosis and risk stratification of myocardial injury. Since its incorporation into the diagnostic criteria for myocardial infarction in 2000, the transition from conventional to high-sensitivity assays has substantially improved the early detection of minor myocardial injury (1, 2). However, this enhanced sensitivity has concurrently increased the frequency of troponin elevations unrelated to acute coronary syndromes (ACS), introducing greater diagnostic complexity.

Among the most challenging scenarios is the elevation of high-sensitivity cardiac troponin T (hs-cTnT) levels in patients with idiopathic inflammatory myopathies (IIM) such as polymyositis (PM), where the troponin elevation may originate from regenerating skeletal muscle rather than the myocardium, representing a true circulating cTnT antigen of non-cardiac origin (3, 4). This diagnostic pitfall can lead to erroneous diagnoses of myocardial infarction and unnecessary invasive procedures.

**Figure 1:**
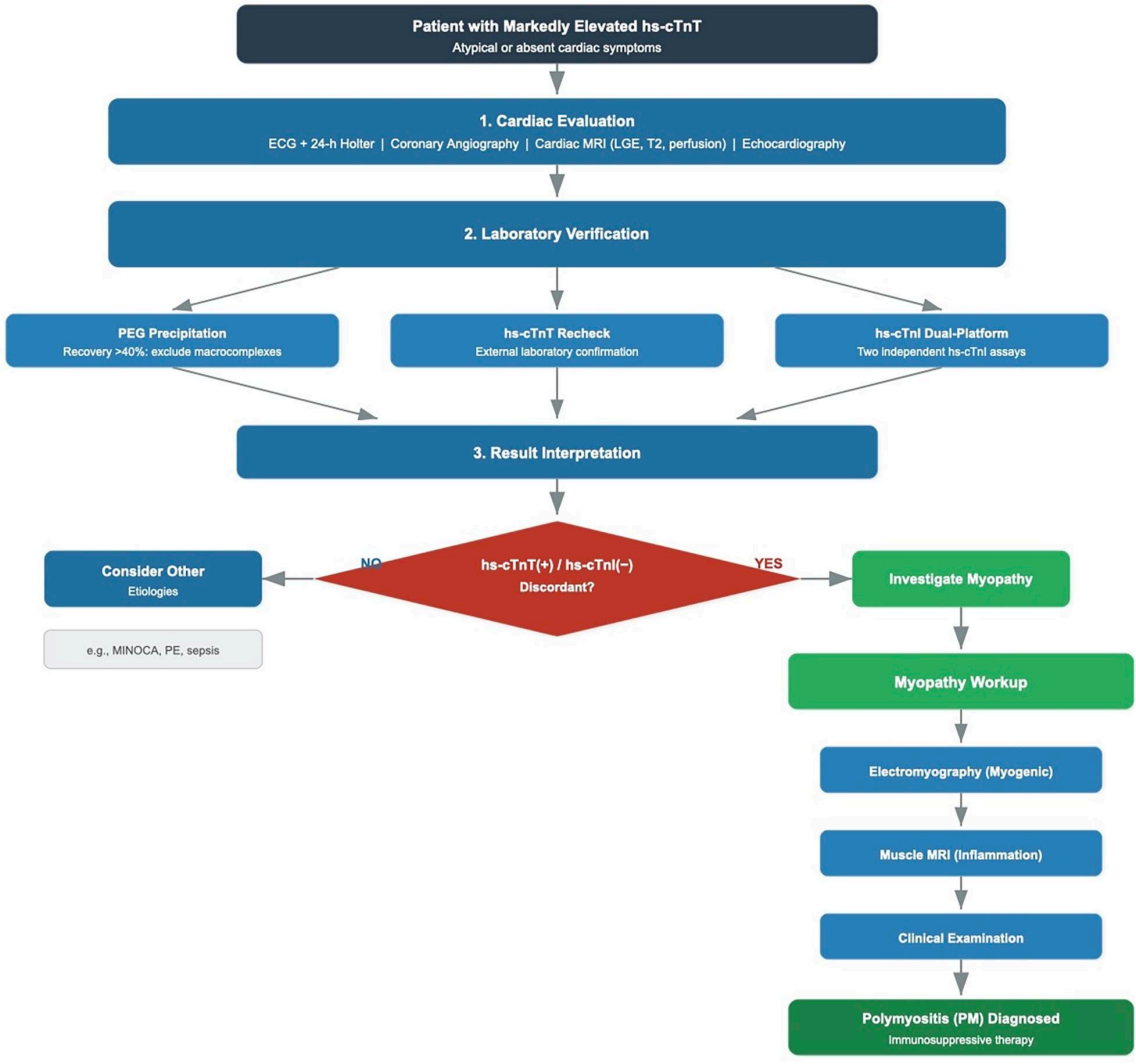
Diagnostic algorithm for the hs-cTnT(+)/hs-cTnI(−) discordance pattern. Diagnostic algorithm for patients with markedly elevated hs-cTnT and atypical or absent cardiac symptoms. Cardiac evaluation (ECG, Holter, coronary angiography, and cardiac MRI) is followed by laboratory verification with PEG precipitation, external hs-cTnT confirmation, and dual-platform hs-cTnI testing. The hs-cTnT(+)/hs-cTnI(−) discordance pattern triggers myopathy workup (EMG, muscle MRI, clinical exam) leading to the diagnosis of polymyositis. hs-cTnT, high-sensitivity cardiac troponin T; hs-cTnI, high-sensitivity cardiac troponin I; PEG, polyethylene glycol; ECG, electrocardiogram; Holter, 24 h ambulatory ECG monitoring; MINOCA, myocardial infarction with non-obstructive coronary arteries; PE, pulmonary embolism.

Herein, we report a case of PM initially suspected of having a non-ST elevation myocardial infarction (NSTEMI) due to markedly elevated hs-cTnT. We describe a systematic laboratory approach—including PEG precipitation, external hs-cTnT verification, and dual-platform hs-cTnI confirmation—to elucidate the true etiology of the troponin elevation. A diagnostic algorithm is proposed to guide clinical decision-making in similar scenarios (Figure 1).

## 2 Case presentation

A 79-year-old female was admitted to our hospital on April 30, 2025, with a one-month history of progressive fatigue and elevated enzymes incidentally discovered during a routine check-up. She had a 20-year history of well-controlled hypertension and diabetes mellitus. There was no history of coronary artery disease, heart failure, or arrhythmia. One month prior to admission, she had received conservative treatment including celecoxib, mecobalamin, and acupuncture for lumbar spondylolisthesis.

The patient denied chest pain, palpitations, dyspnea, fever, or proximal muscle pain. Physical examination revealed stable vital signs and full consciousness. Neurological examination demonstrated muscle strength of 4/5 in all four extremities with normal muscle tone and no lower extremity edema.

The patient's initial cardiac enzyme profile (Beckman Coulter, USA), tested at our outpatient clinic, showed the following results: lactate dehydrogenase (LDH) 1,145.4 U/L (reference 120–250 U/L), creatine kinase (CK) 6,906.4 U/L (reference 40–200 U/L), creatine kinase muscle/brain (CK-MB) 342.7 U/L (reference 0–19 U/L). Concurrently, cardiac injury biomarkers (Roche, Switzerland) showed the following results: myoglobin (MYO) 2,605 ng/mL (reference 25–58 ng/mL), and hs-cTnT 521 ng/L (reference 0–14 ng/L).

The initial 12-lead electrocardiogram (ECG) showed sinus rhythm with non-specific ST-T segment changes in the lateral leads. No ST-segment elevation, pathological Q waves, or dynamic T-wave inversion suggestive of acute ischemia were observed. Twenty-four-hour Holter monitoring (24 h ambulatory ECG monitoring) demonstrated frequent atrial premature beats (1,789/24 h) and incomplete right bundle branch block. Heart rate variability was mildly to moderately reduced (SDNN 81 ms, DC 3.24 ms), potentially reflecting early cardiac autonomic involvement.

While these non-specific repolarization abnormalities were not diagnostic of acute coronary syndrome, they were considered potentially compatible with an atypical NSTEMI presentation in the context of the markedly elevated hs-cTnT. Emergency coronary angiography revealed only mild to moderate coronary stenosis with patent blood flow (TIMI grade 3) and no evidence of acute thrombotic occlusion. Coronary physiology testing (e.g., coronary flow reserve assessment) and vasomotor provocation (e.g., acetylcholine testing) were not performed; therefore, myocardial infarction with non-obstructive coronary arteries (MINOCA) due to coronary microvascular dysfunction or vasospasm cannot be definitively excluded. Concurrently, hydration therapy was initiated to prevent potential acute kidney injury from rhabdomyolysis.

Additional laboratory examinations on admission revealed mild neutrophilic leukocytosis: white blood cell count 9.85 × 10^9^/L (reference 3.5–9.5 × 10^9^/L), neutrophilia (neutrophils 6.66 × 10^9^/L, reference 1.8–6.3 × 10^9^/L) and a normal lymphocyte count. Hepatic function panels showed markedly elevated transaminases: alanine aminotransferase (ALT) 360.6 U/L (reference 7–40 U/L) and aspartate aminotransferase (AST) 333.1 U/L (reference 13–35 U/L). High-sensitivity C-reactive protein (hs-CRP) was within normal limits at 1.27 mg/L (reference 0–3 mg/L); and HbA1c was 7.6% (reference 4%–6.5%). Renal function, lipid profile, vasculitis antibodies, antinuclear antibody (ANA), rheumatoid factor, complement C3, C4, and erythrocyte sedimentation rate (ESR) were all within normal limits. Chest computed tomography (CT) revealed scattered patchy and nodular opacities in both lungs with interstitial changes. Abdominal CT and echocardiography showed no significant abnormalities.

Despite the absence of cardiac symptoms and ongoing hydration therapy, hs-cTnT levels remained persistently elevated (>300 ng/L) throughout hospitalization (Table 1). On day 6, a laboratory consultation was requested, and hs-cTnT was rechecked at an external hospital (Roche, Switzerland), confirming the elevation. PEG precipitation was subsequently performed on three samples collected at different time points, yielding recovery rates of 84.0%, 81.8%, and 86.3%, respectively (Table 2). These high recovery rates effectively ruled out interference from macromolecular troponin complexes or heterophile antibodies, confirming the presence of genuine circulating cTnT antigen.

**Table 1:** Dynamic changes in serum biomarkers during hospitalization.

Date (2025)	AST (U/L)	LDH (U/L)	CK (U/L)	CK-MB (U/L)	MYO (ng/mL)	hs-CTNT (ng/L)
	13–35	120–250	40–200	0–19	25–58	0–14
Apr 30	333.1	1,145.4	6,906.4	342.4	2,605	521
May 01	—	—	4,954.3	221.6	1,685	—
May 02	257.3	869.2	4,805.3	248.3	—	—
May 03	224.7	843.9	4,154.2	219.2	1,518	369
May 04	217.8	743.3	4,182.7	221.4	1,743	413
May 06	227.9	783.5	4,634.3	228.8	1,772	422
May 08	246.6	1,009.7	5,117.2	264	1,970	467
May 12	214.2	894.5	4,829.8	250.1	1,644	471

Reference ranges: AST, 13–35 U/L; LDH, 120–250 U/L; CK, 40–200 U/L; CK-MB, 0–19 U/L; MYO, 25–58 ng/mL; hs-cTnT, 0–14 ng/L.

**Table 2:** Results of PEG precipitation analysis for hs-cTnT.

Pre-PEG	Post-PEG^a^	Recovery rate^b^
ng/L	ng/L	%^c^
369	310	84.0
413	338	81.8
422	364	86.3

a Post-PEG concentrations (conc.) are corrected for the dilution factor (1:1).

b Recovery rate = (Post-PEG conc./Pre-PEG conc.) × 100%.

c A recovery rate <40% usually indicates macromolecular antibody interference, while a rate >60–80% suggests monomeric troponin (true elevation).

In parallel, serum samples were sent to two independent reference laboratories for hs-cTnI analysis using the Abbott (USA) and YHLO (China) platforms. Both assays returned results within the normal reference range, establishing a clear hs-cTnT(+)/hs-cTnI(−) discordance pattern.

On day 8 of hospitalization, cardiac magnetic resonance (CMR) imaging was performed. Cine sequences demonstrated preserved left ventricular systolic function (ejection fraction 61%) and coordinated wall motion. T2-weighted imaging showed no myocardial edema, and resting perfusion was unremarkable. Late gadolinium enhancement (LGE) imaging, however, revealed a few linear areas of hyperenhancement in the lateral left ventricular wall with focal epicardial thickening and enhancement. A small pericardial effusion was also noted. These CMR findings suggest possible subclinical myocardial involvement in the form of focal myocarditis or epicardial inflammation related to polymyositis; however, the absence of T2 edema and perfusion abnormalities, combined with persistently normal hs-cTnI levels, argues against active myocardial necrosis as the primary source of the elevated hs-cTnT.

The isolated elevation of hs-cTnT, in the presence of persistently normal hs-cTnI, strongly indicated a skeletal muscle origin. Subsequent electromyography (EMG) revealed myogenic damage, and physical examination identified masticatory muscle involvement.

Based on these integrated findings, a final diagnosis of polymyositis (PM) was established. The patient was subsequently transferred to the rheumatology department of another hospital for specialized management, where muscle MRI further demonstrated features consistent with inflammatory myopathy. Immunosuppressive therapy (glucocorticoids and intravenous immunoglobulin) was initiated, resulting in clinical improvement with progressive recovery of muscle strength. Given the primary diagnostic focus of this report, detailed treatment protocols and precise biochemical normalization timelines from the referring hospital are not available.

## 3 Discussion

Creatine kinase (CK), CK-MB, and cardiac troponins are established biomarkers for myocardial ischemia. The troponin complex (I, T, and C) regulates calcium-mediated contraction through tissue-specific isoforms; notably, cTnI and cTnT are encoded by genes distinct from their skeletal muscle counterparts (sTnI and sTnT), whereas cTnC is shared with slow-twitch skeletal muscle and is therefore unsuitable for cardiac diagnosis (4–6). With the advent of high-sensitivity assays, hs-cTnT and hs-cTnI have substantially improved the detection of minor myocardial injury but have concurrently increased the frequency of non-ischemic elevations encountered in clinical practice. Among the most diagnostically challenging scenarios is the elevation of hs-cTnT in patients with IIM, where the circulating cTnT may represent a true antigen of skeletal muscle origin rather than an analytical artifact—a pitfall that can lead to unnecessary invasive procedures if not correctly interpreted (5, 7, 8).

Historically, cTnT was considered an absolutely cardiac-specific antigen. However, in chronic active myopathies, skeletal muscle regeneration can upregulate *TNNT2* gene expression, leading to re-expression of embryonic cTnT isoforms (9). These isoforms exhibit structural homology with cardiac cTnT and cross-react in commercial high-sensitivity immunoassays. A recent review confirmed that hs-cTnT is elevated in 45%–69% of patients with skeletal muscle disorders, while hs-cTnI remains largely unaffected (8). Consequently, what appears as a “false-positive” is, in fact, authentic monomeric cTnT released from regenerating skeletal muscle—a “troponin trap” that mimics myocardial injury (7, 10).

In contrast, cTnI exhibits markedly stricter tissue specificity. The cardiac isoform is transiently co-expressed with slow skeletal muscle TnI during the first 9 months after birth, after which its expression becomes strictly confined to the myocardium (5); there is no evidence of cTnI re-expression during skeletal muscle regeneration or repair (10–12). When genuinely elevated, cTnI is significantly associated with active IIM and serves as a robust predictor of adverse prognosis (13, 14). The discordance between elevated hs-cTnT and persistently normal hs-cTnI therefore represents a key diagnostic discriminator indicating a skeletal muscle origin of the circulating cTnT.

In the present case, PEG precipitation yielded recovery rates consistently exceeding 80%, effectively excluding interference from macro-troponin complexes and heterophile antibodies and confirming that the hs-cTnT signal represented authentic monomeric cTnT antigen. This finding redirected the diagnostic focus from analytical interference toward a biological source. Although the markedly elevated hs-cTnT and non-specific ECG changes initially raised suspicion of atypical ACS, the absence of canonical cardiac symptoms, progressive muscle weakness, and normal coronary angiography argued against acute myocardial ischemia.

The incorporation of CMR provided critical pathophysiological insights. The presence of non-transmural LGE in the lateral left ventricular wall without accompanied T2-weighted edema or perfusion abnormalities suggested subclinical epicardial inflammation or chronic, non-necrotic myocardial involvement, which is known to occur in 9%–72% of IIM patients (13). Crucially, the persistently normal hs-cTnI confirmed on two independent high-sensitivity platforms strongly supported the conclusion that active myocardial necrosis was not the primary source of the markedly elevated hs-cTnT. Instead, the circulating hs-cTnT was predominantly derived from the severely damaged and regenerating skeletal muscle mass, as evidenced by the extreme elevations in total CK (6,906.4 U/L) and CK-MB (342.7 U/L).

Similar to hs-cTnT, CK-MB can be markedly elevated in IIM due to chronic skeletal muscle regeneration, with concurrent elevation of the CK-MB fraction and CK-MB/total CK ratio (10, 15). In PM patients, cTnT levels correlate strongly with CK-MB, whereas hs-cTnI remains unaffected unless concurrent myocarditis is present (4). Our findings reinforce that when managing patients with systemic myopathies, hs-cTnI—rather than hs-cTnT or CK-MB—must be utilized as the definitive biomarker to guide the evaluation of acute cardiac injury.

## 4 Conclusion

In summary, skeletal muscle regeneration can trigger the re-expression of embryonic cTnT isoforms, producing a true circulating cTnT elevation of skeletal muscle origin that mimics myocardial injury. The hs-cTnT(+)/hs-cTnI(−) discordance pattern, confirmed by PEG precipitation to exclude analytical interference, is the key diagnostic discriminator in this setting. Clinicians encountering markedly elevated hs-cTnT with normal hs-cTnI, particularly in patients with atypical or absent cardiac symptoms, should prioritize investigation for underlying myopathy (Figure 1) rather than pursuing unnecessary antithrombotic therapy or invasive coronary angiography.

Several limitations of this case report should be acknowledged. First, although cardiac magnetic resonance imaging was performed and revealed possible subclinical myocardial involvement, the absence of T2-weighted edema and resting perfusion abnormalities, combined with persistently normal hs-cTnI on two independent platforms, strongly argues against active myocardial necrosis as the primary source of the hs-cTnT elevation. Nevertheless, the possibility of subclinical myocarditis or epicardial inflammation related to polymyositis cannot be fully excluded without histopathological confirmation. Second, coronary physiology testing and vasomotor provocation were not performed at the time of coronary angiography; therefore, MINOCA cannot be categorically excluded. Third, the 24 h Holter monitoring demonstrated mildly to moderately reduced heart rate variability, which may reflect early cardiac autonomic involvement. Fourth, detailed treatment data and biochemical normalization timelines are unavailable as the patient received specialized rheumatologic care at another institution; however, this does not affect the primary diagnostic conclusions of this report.

Despite these limitations, the markedly elevated skeletal muscle enzymes, the hs-cTnT(+)/hs-cTnI(−) discordance pattern, the PEG precipitation recovery rates, and the clinical response to immunosuppressive therapy collectively support a skeletal muscle origin as the predominant contributor to the hs-cTnT elevation.

## Data Availability

The original contributions presented in the study are included in the article/Supplementary Material, further inquiries can be directed to the corresponding author/s.
